# Preparation of European Public Health Professionals in the Twenty-first Century

**DOI:** 10.3389/fpubh.2017.00018

**Published:** 2017-02-15

**Authors:** Vesna Bjegovic-Mikanovic, Robert Otok

**Affiliations:** ^1^Faculty of Medicine, Belgrade University, Belgrade, Serbia; ^2^Association of Schools of Public Health in the European Region (ASPHER), Brussels, Belgium

**Keywords:** public health, education, professionals, partnerships, Europe

## Abstract

The public health profession in Europe has a leadership role for ensuring European’s health in the twenty-first century and therefore must assume responsibility for advancing education for research and practice. Three fundamental questions are explored: (1) What are the main public health problems facing public health professionals; (2) What are their existing competencies after training; and (3) What competencies do European employers expect? The European Schools of Public Health assessed their best success to be in the field of health promotion, followed by disease prevention including identification of priority health problems, and elimination of health hazards in the community. Conversely, they see the least success in dealing with preparedness and planning for public health emergencies. From an employer’s perspective, significant gaps between current and desired levels of performance at the job exist for all Essential Public Health Operations of World Health Organization. Based on prior research and recent European surveys of Schools and Departments of Public Health, the following recommendations are made, which emphasize the leadership role of the European public health community: (1) the preparation of public health professionals requires an interface between public health functions, competencies, and performance; (2) competence-based education is important and allows debates on the scope of the required education; (3) governments have to realize that the present lack of infrastructure and capacity is detrimental to the people’s health; (4) as public health challenges are increasingly global, educational institutions have to look beyond the national boundaries and participate in European and global networks for education, research, and practice.

## Introduction

Today public health professionals face many new and demanding challenges such as how to increase healthy life expectancy and to minimize health inequalities in times of repeated financial crises. Equity in health across the European region and beyond is viewed as one of the central goals of sustainable development and dedicated work of public health professionals to achieve improvements in planetary health (1, 2). To cope effectively with these challenges, it is essential to address the multiple socioeconomic, environmental, and individual determinants of health. This requires that the public health profession assumes leadership in the twenty-first century and understands that “working differently means leading and learning differently” (3).

This perspective article starts out with a description of the European landscape of professional education including reference to US American developments. Against this background, we try to find answers to three fundamental questions based on the published literature and the European survey of Schools and Departments of Public Health (SDPHs) (4):

(1) What are the main public health problems that we have to address in Europe and beyond?
(2) What are presently the exiting competences of public health professionals after training in Europe?
(3) What are the expectations of European employers of public health professionals?

## The European Landscape of Professional Education

Globally and also within the European Region with its 53 countries, public health professionals are confronted with an extremely heterogeneous landscape. Nevertheless, the European public health education and training has the same cornerstones and develops along the Bologna Process, which is designed to harmonize the fragmented educational scene in Europe and to facilitate the international exchange of students and lecturers. Meanwhile, almost all European countries have fully joined in promoting the attractiveness of European Higher Education Area (5). The degree structure is now based either on two or three of the major strata—bachelor, master, and doctoral level (6). Application of the European Credit Transfer System (ECTS)^1^ in academic programs is the primary instrument for the mutual recognition of diplomas and professional mobility. Recent challenges lead to the development of student-centered learning, modularization, focus on learning outcomes, and on applications for lifelong learning (7).

Despite many different educational backgrounds of the current public health workforce, consensus is emerging to focus on three main layers (8): (1) public health professionals; (2) health professionals; and (3) other professionals with job functions bearing on the population’s health. Also, understanding of the different job settings is of great importance for accountable performance (9). A European set of competencies has been developed led by the Association of Schools of Public Health in the European Region (ASPHER) (10) and adopted by the World Health Organization (WHO) (11). The European competencies comprise six domains (abbreviated) (12): (1) methods in public health; (2) population health—its social and economic determinants; (3) population health—its material environmental determinants; (4) policy, economics, organization, management, and leadership; (5) health promotion, health education, health protection, and disease prevention; and (6) ethics. US American competence frameworks have also been discussed for many years (13). In 2014, the Council on Linkages Between Academia and Public Health Practice published a very differentiated set of competencies (14) organized according to eight domains. In spite of the different systems and wording, the contents are quite similar, especially in respect to an outcome-based education and the apparent need for interprofessional education and lifelong learning.

## Exploration of Key Questions

### What Are the Main Public Health Problems That We Have to Address in Europe and Beyond?

The competence acquired during the public health education has to support qualified performance to solve dominating (global) public health problems as there are threats to humanity (15) and planetary health (1) coming with climate change (e.g., floods, desertification), social inequity (e.g., poverty, hunger), insecurity (e.g., armed conflicts, terrorism), and instability (e.g., financial crises).

To some degree, public health functions and services are regionally specific (11, 16, 17), nevertheless in its document “Health Workforce 2030” WHO underlines the unmet global demand for qualified health professionals including the public health workforce (18). By now, public health interventions in all countries target predominantly socioeconomic factors (19). One of the most significant public health challenges—well-being—has attracted the particular interest of public health professionals. It represents one of the most complete and profound reflections of health. In the new European health policy “Health 2020,” signed and adopted by 53 member states of the Region in September 2012, WHO stated that the aim is to improve the health and well-being of populations significantly (20). Moreover, in September 2015, 193 countries that are members of the United Nations signed the “2030 Agenda for Sustainable Development”; Goal 3 is specific: “to ensure healthy lives and promote well-being for all at all ages” (21).

### What Are the Exiting Competences of Public Health Professionals after Training in Europe?

For this and the third question, we refer mainly to the unique regionwide surveys organized and published on behalf of ASPHER, “*the key independent European organisation dedicated to strengthening the role of public health by improving education and training of public health professionals for both practice and research*” (22). WHO Europe invited ASPHER to take the lead on the Essential Public Health Operation (EPHO) No. 7 on “Assuring a sufficient and competent public health workforce” (23).

Looking at the performance of public health professionals, while there are different published models of health system performance (24–26), very few of them exist in the field of public health and very often relate exclusively to the concept of management (27). ASPHER’s approach assumes that performance varies and strives for success in achieving long-term objectives. Therefore, all four dimensions of the WHO performance model should be included in a comprehensive assessment of public health performance in the future (25):

- *Availability* regarding space and time and the distribution and attendance of existing workers;
- *Competence* expressed as the combination of technical knowledge, skills, and behaviors;
- *Responsivenes*s related to people who are treated decently, regardless of whether or not their health improves;
- *Productivity* connected to maximum effective public health services and health, reducing waste of staff time or skills.

The Global Independent Commission on Education of Health Professionals for the twenty-first century stated that vocational education had not kept pace with the new global challenges, largely because of fragmented, outdated, and static curricula that produce ill-equipped graduates (28). ASPHER (29) reacted by executing in 2011–2012 the first systematic survey of, at that time, 81 members, SDPHs. With a participation rate of 81.5%, the survey included the evaluation of exiting competencies of their Master of Public Health (MPH) graduates making use of the European list of public health competencies (10) and the EPHOs, endorsed by WHO (11).

Together more than 80 different master programs in the broader field of public health are offered in Europe. Although epidemiology maintains the first rank in a list of curricular contents, critical new areas like informatics, genomics, community-based participatory research, policy and law, global health, and ethics are also present. One major focus of the survey was the dimension of competencies for good public health performance (29). SDPHs assessed their best success to be in the field of health promotion, followed by disease prevention including identification of priority health problems, and health hazards in the community while they see the least success in dealing with preparedness and planning for public health emergencies.

However, European training capacities are entirely insufficient, even taking into consideration that not all MPH training programs in Europe are based on schools of public health and are not covered by the ASPHER survey. Their role in the development of the public health workforce warrants separate consideration. The ASPHER survey found an overall average of 46 graduates per institution per year (all programs of the Bologna levels and equivalents). This total annual number of 3,035 graduates by the institutions participating in the survey can by no means satisfy the need for public health professionals which for the European Union alone can be estimated to be 22,000 based on levels proposed for the United States (30, 31).

Given an accelerated globalization and the impact of developments outside of Europe, training for global public health has rapidly gained relevance. Global health is present in the curricula of 82% of ASPHER members with a median of 40 h, only a bit higher than recommended for medical students as a minimum (30 h) (32).

### What Are the Expectations of European Employers of Public Health Professionals?

The ASPHER survey 2012–2013 identifies in addition to exit competencies of graduates also the current as well as the desired performance of public health professionals as determined by 63 out of 109 (57.8%) contacted European employers (33). Employers were asked: “*How often public health professionals in your job environment perform the task, which requires selected competence*?” Furthermore, they had to specify the current level of performance the desired level. It turned out that the regular performance of a particular competence is defined by what is “necessary” and driven by assigned tasks. Employed professionals rarely have occasion to perform a particular public health competence on an everyday basis or weekly. Significant gaps exist between current and desired levels of performance on the job for all EPHOs (4).

**Figure 1:**
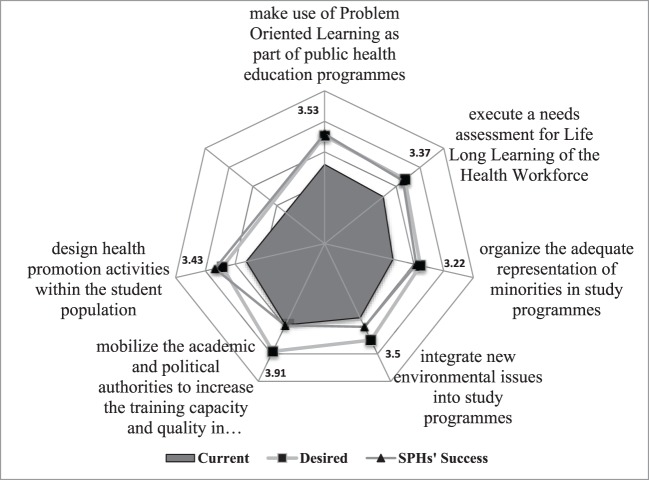
**Essential Public Health Operation (EPHO) 7: assuring a competent public health and personal healthcare workforce**. [The rays in the spider web indicate the ranks in a 5-point Likert scale (3 = average, 5 = best) for the six competences selected to represent EPHO 7.] Source: ASPHER WG on Innovation and Good Practice in Public Health Education (4).

Schools and Departments of Public Health estimates of their success in terms of the public health exiting competencies of their graduates often are not in congruence with employers’ estimates of current levels of performance of their staff (see the representative example of EPHO 7 with its related competencies in Figure 1). This observation can be due to a favorable overestimation by SDPHs or a generational representation in the sense that the current employees do not perform accordingly because they were educated in earlier times. In most areas however, SPHDs’ opinion about their present success corresponds quite closely to desired levels of performance as stated by employers. The main exceptions as published (33) are EPHOs related to preparedness and planning for public health emergencies, health protection operations, and core communication for public health. SPHDs estimate their success considerably lower in those EPHOs than public health employers expect as desired level of performance. A way to improve this deficit could be to involve public and private employers already in the curriculum development.

## Networking and Partnerships in Preparing Public Health Professionals

The answers we found to the three questions discussed can be summarized as follows:

(1) Increasingly, Europe has to confront threats related to the rapid globalization and their upstream determinants, various strategies for improvement are formulated and adopted by the United Nations and the WHO. New research fields have been introduced like well-being.
(2) In spite of the high variation of the institutional format, there is considerable agreement on what kind and level of knowledge and skills are required and provided to equip public health graduates for a successful professional career. Nevertheless, the training capacity in public health is entirely insufficient, especially concerning global health and emergency preparedness.
(3) Potential employers of public health are in need of specific competencies to perform a specific task at a specific moment. In consequence, the master programs and even more continuing education have to allow for high flexibility responding to the requests of students and potential employers.

Given the small infrastructural base, the logical way forward is the advancement of partnerships joining capacities at the European and global level (32). Today learning from public health experience in other countries by including international students in the program and fostering mobility becomes increasingly important. Various forms of regional collaboration are improving outcomes in public health education and practice.

An example is the intergovernmental South-Eastern Europe Health Network (SEEHN) (34) of WHO Europe and in the recent past the Forum for Public Health Education, Training and Research in Southeastern Europe (35), supported by the European Stability Pact. Examples of such cooperation are common sets of flexible teaching materials which in the meantime comprise six thematic volumes with close to 250 modules on some 4,000 pages and the open-access South Eastern European Journal of Public Health (36), which also offers the second edition of the mentioned teaching books (37, 38).

This experience represents not only a valuable support for students and faculties but also has served to establish close collaboration across borders and among people who have been on different sides during the wars of the 90s. As an example, to the volume on Management in Health Care Practice, published 2008, 49 authors from 10 countries have contributed. There were many occasions to test teaching modules as applied (39) and learn through conferences/meetings, summer/winter schools, and students’ conferences during a decade of cooperation.

Also, a joint project of the Open Society Foundations and ASPHER facilitated the establishment of schools of public health in selected countries of Southeastern Europe, with the primary focus on the development of teaching curricula at Masters’ level in the field of public health sciences (40).

Schools of Public Health, Institutes of Public Health, and National Public Health Association have established a permanent collaboration to improve the performance of public health professionals. For example, ASPHER (7) and the European Public Health Association (41) are both regional members of the World Federation of Public Health Associations (WFPHA) (42), whereas the International Association of National Public Health Institutes is a member of the Advisory Board of WFPHA.

## Recommendations

The authors recommend the following to prepare a competent public health leadership in Europe:

1. Preparing public health professionals requires an interface between public health functions, competencies, and performance. Analyzing the existing situation and planning for future needs, education and research are core composite parts in advancing a strong public health profession. Increasing cross-border mobility stimulates the higher educational institutions to become transnational actors.
2. Competence-based education is important and opens debates on the scope of the required education. Based on the recent ASPHER survey, the European schools are still on their way preparing public health professionals to perform at highest levels.
3. Governments have to realize that investing in the public’s health workforce bears high returns. The present lack of infrastructure and capacity is detrimental to the people’s health.
4. As public health challenges are increasingly global, educational institutions have to look beyond the national boundaries and participate in European and global networks for education, research, and practice.

## Author Contributions

VB-M: conceptualization, introduction, sections 2 and 3, section on networking. RO: section on European landscape, section 1. VB-M and RO: recommendations.

## Conflict of Interest Statement

The authors declare that the research was conducted in the absence of any commercial or financial relationships that could be construed as a potential conflict of interest.

## Footnotes

^1^One credit point of ECTS corresponds to 25–30 h of student’s workload, thereof up to 30% in direct contact with a lecturer (through an interactive presentation, exercise, seminar, field work, or research).
